# DNA methylation of silencer-annotated regions at the *MGMT* locus in the context of promoter and enhancer methylation in glioblastoma and melanoma brain metastases

**DOI:** 10.1186/s13148-026-02204-y

**Published:** 2026-08-01

**Authors:** Katharina Pühringer, Fabian Firlinger, Benno Fehringer, Serge Weis, Sabine Spiegl-Kreinecker, Margit Cichna-Markl

**Affiliations:** 1 Institute of Analytical Chemistry, Faculty of Chemistry, University of Vienna, Vienna, Austria; 2 Vienna Doctoral School in Chemistry (DoSChem), University of Vienna, 1090 Vienna, Austria; 3 Division of Neuropathology, Department of Pathology and Molecular Pathology, Kepler University Hospital GmbH, Johannes Kepler University, Linz, Austria; 4 Department of Neurosurgery, Johannes Kepler University, Kepler University Hospital GmbH, Linz, Austria; 5 Clinical Research Institute for Neurosciences, Johannes Kepler University, Linz, Austria

## Abstract

**Background:**

*MGMT* promoter methylation is a well-established biomarker in glioblastoma, its relevance in melanoma brain metastases remains controversial. Recent studies indicate that methylation of *MGMT* enhancers contributes to *MGMT* regulation, whereas the role of silencer methylation has not yet been investigated.

**Methods:**

DNA methylation of 52 CpG sites was analyzed in regions annotated as potential silencers located in the promoter, intronic, and upstream regions of *MGMT*. These regions were selected based on genomic annotations and their genomic proximity to *MGMT*, encompassing sites to approximately 1.5 Mb upstream, without experimental validation of direct regulatory activity. Analyses were performed in glioblastoma and melanoma brain metastases using in-house developed assays based on bisulfite conversion, PCR amplification of target regions, and pyrosequencing. By integrating previously published promoter and enhancer methylation data, a DNA methylation landscape of *MGMT* regulatory elements was established. Associations between CpG methylation and MGMT protein expression, overall survival, patient age, and sex were evaluated.

**Results:**

The degree of methylation strongly depended on the genomic location of the respective regulatory element. Both shared and tumor-type-specific methylation patterns were observed in glioblastoma and melanoma brain metastases. Promoter-associated CpGs and selected intronic silencer-annotated regions showed associations with MGMT protein expression. In glioblastoma, several promoter-associated CpGs were associated with overall survival, whereas sex-specific and age-related effects were absent.

**Conclusions:**

DNA methylation of *MGMT* regulatory elements is highly CpG-specific. CpG-resolved analyses reveal a complex epigenetic landscape across the *MGMT* locus and highlight substantial regional heterogeneity. These findings suggest that extending methylation analyses beyond the classical promoter region provides additional insights into *MGMT*-associated epigenetic variation and its potentical clinical associations.

**Supplementary Information:**

The online version contains supplementary material available at https://doi.org/10.1186/s13148-026-02204-y.

## Introduction

O6-Methylguanine-DNA methyltransferase (MGMT) is a key DNA repair enzyme that protects cells from the cytotoxic effects of alkylating agents by removing alkylating lesions from the O6 position of guanine [1]. MGMT expression is primarily regulated at the epigenetic level, predominantly through DNA methylation of its promoter [2], with promoter methylation generally resulting in transcriptional silencing [3].

The level of MGMT expression in a tumor has a major impact on the efficacy of treatment with alkylating agents such as temozolomide (TMZ) [4]. High MGMT levels are frequently associated with treatment failure and drug resistance [5]. Since clinical trials demonstrated that *MGMT* promoter methylation is associated with a survival benefit from TMZ therapy in glioblastoma (GB) [6–9], *MGMT* promoter methylation has emerged as a predictive biomarker of TMZ in GB patients [10]. In addition, several studies have reported its suitability as a prognostic biomarker in GB [11, 12].

However, a substantial subset of GB cases exhibits discordance between *MGMT* promoter methylation status and MGMT expression levels [13]. Tumors with a methylated promoter may retain measurable MGMT expression, whereas others with an unmethylated promoter show unexpectedly low expression [14, 15]. These findings suggest that promoter methylation alone does not fully explain *MGMT* regulation and that additional regulatory mechanisms contribute to the control of *MGMT* transcription [15].

Recent studies have demonstrated that DNA methylation of distal enhancer regions is linked to *MGMT* promoter methylation and protein expression [16, 17]. Moreover, the methylation status of individual CpGs within *MGMT* enhancers was associated with common genetic variants and clinical parameters of the patients, including overall survival [17, 18]. A very recent study further identified both shared and tumor-specific methylation patterns of the *MGMT* promoter and enhancers in GB and melanoma brain metastases (MBM), suggesting that *MGMT* epigenetic regulation is complex and context dependent [19].

Although silencers are important modulators of gene expression [20, 21], the role of DNA methylation in silencer-annotated regions associated with the *MGMT* locus has not been investigated.

The aim of this study was therefore to investigate whether DNA methylation patterns in silencer-annotated regions within and surrounding the *MGMT* locus, including distal regions located up to approximately 1.5 Mb upstream of *MGMT*, complement existing information on promoter and enhancer methylation. By integrating data from enhancers and the promoter, we aimed to generate a DNA methylation landscape of *MGMT* regulatory elements, essential for better understanding the epigenetic mechanisms controlling MGMT expression and their potential clinical implications.

## Materials and methods

### Samples and cell culturing

Approval for this study was granted by the Ethics Commission of the Faculty of Medicine at Johannes Kepler University Linz (application number E-39-15). All patients signed written informed consent forms prior to participation. GB and MBM samples originate from the Department of Neurosurgery, Kepler University Hospital Linz, where patients underwent surgery between 2001 and 2020. Cohort tables with relevant clinical parameters and covariates are provided in Supplementary Table S1 and S2. All analyses were performed on immortalized cell cultures established from surgically resected tumor tissues. Cell culturing, harvesting and storage was carried out as described previously [22]. In all cases, low-passage immortalized cell lines were used. Previous analyses of matched primary tumor tissues and corresponding immortalized cell cultures demonstrated a high concordance of DNA methylation patterns and provided no evidence of epigenetic drift [19].

### MGMT expression

For Western blot analysis, protein extracts were separated by SDS-PAGE, transferred onto PVDF membranes, and incubated with the monoclonal mouse antibodies anti-human MGMT (clone MT3.1; Merck Millipore, Darmstadt, Germany) and anti-β-Actin (clone AC-15; Sigma-Aldrich, St. Louis, MO, USA). Immunoreactive bands were detected using the ECL Prime Western Blotting Detection Reagent (Cytiva, Marlborough, MA, USA) according to the manufacturer’s instructions and visualized with the ChemiDoc Imaging System (Bio-Rad, Hercules, CA, USA). β-Actin, major component of the cytoskeleton and present at relatively high levels in most eukaryotic cells, served as a loading control to verify equal protein loading across samples. MGMT protein expression was quantified and normalized to β-Actin levels.

*MGMT* mRNA expression was determined by RT-PCR as previously described [22]. PCR products were separated by polyacrylamide gel electrophoresis, stained with GelRed Nucleic Acid Gel Stain, and quantified by densitometric analysis using the ChemiDoc Imaging System and Image Lab software (Bio-Rad, Hercules, CA, USA). Expression levels were normalized to *GAPDH* mRNA, which served as an endogenous reference gene and was co-amplified in parallel.

### DNA extraction and bisulfite conversion

DNA extraction from pelleted cells and bisulfite conversion were performed using QIAamp DNA Blood Mini Kit (Qiagen, Hilden, Germany) and EpiTect Fast Bisulfite Conversion Kit (Qiagen, Hilden, Germany) according to the manufacturers’ instructions, as described previously [17]. The Qubit 4 instrument was used for DNA concentration measurement (using Qubit dsDNA BR Assay Kit for genomic and Qubit ssDNA Assay Kit for bisulfite converted DNA (both from Thermo Scientific, Vienna, Austria)).

### Primer design and DNA methylation analysis

**Fig. 1:**
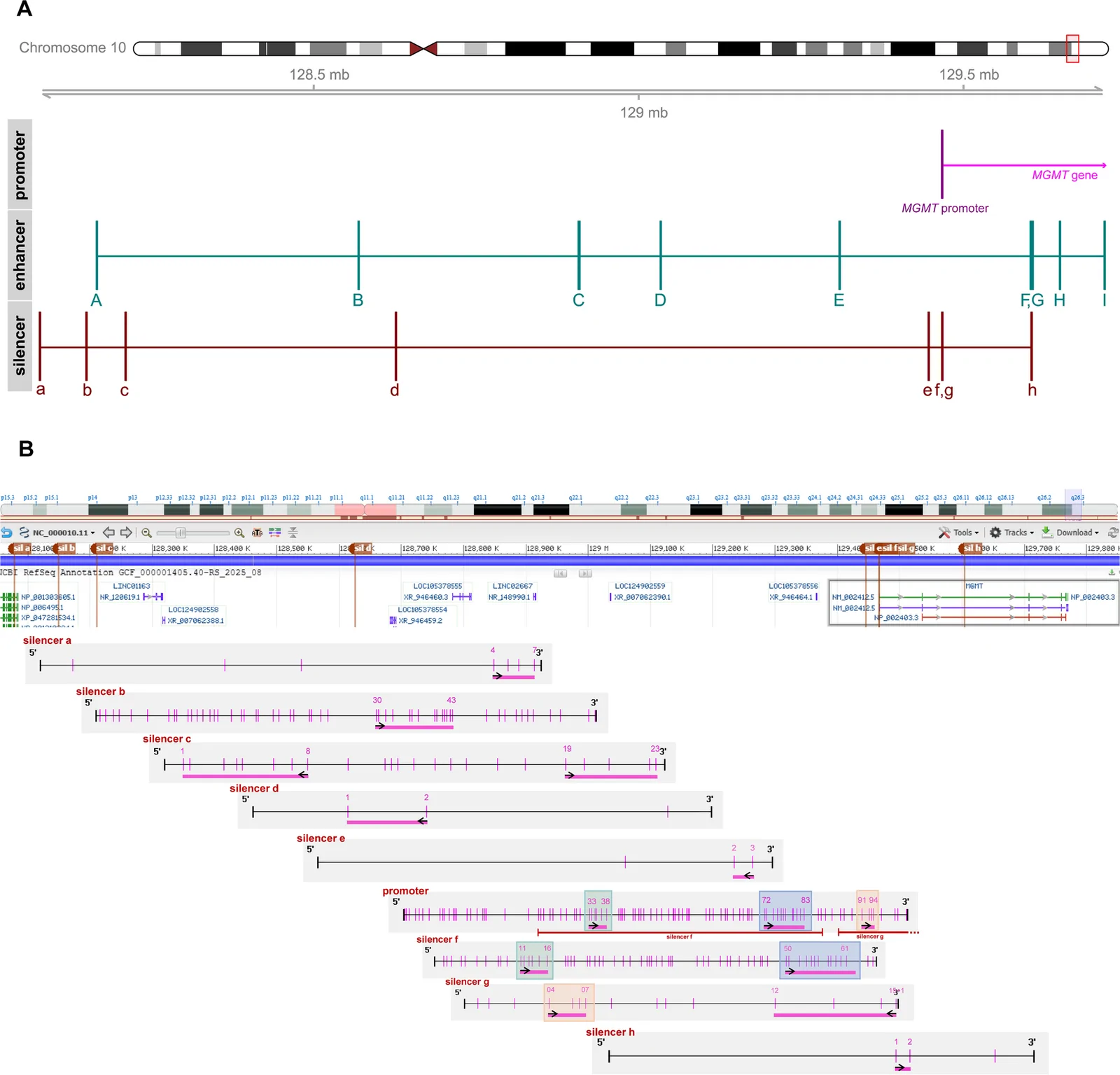
Genomic location of *MGMT* regulatory elements and CpGs targeted in this study. **A** Positions of the *MGMT* promoter, enhancers, and silencer-annotated regions on chromosome 10. **B** Overview of designed assays targeting CpGs within silencer-annotated regions. Assays and CpG sites are represented by pink bars and vertical lines, respectively. Black arrows indicate the sequencing direction. CpG sites in intergenic silencers a–e are labelled Sa–Se and numbered according to their position within each silencer. CpG sites in promoter-associated silencers are labelled PS and numbered according to their position within the *MGMT* promoter. PS-33–PS-38 and PS-72–PS-83 correspond to CpG sites 11–16 and 50–61 in silencer f, respectively. PS-91–PS-94 correspond to CpG sites 04–07 in silencer g. The CpG sites PS-72–PS-83 represent the promoter region commonly used as a predictive biomarker in GB. CpG sites in intron-associated silencers g and h are labelled Sg and Sh and numbered according to their position within the respective silencer. Graphical CpG representation was generated using Methyl Primer Express Software v1.0 (Thermo Scientific). Chromosome 10 coordinates and the location of the *MGMT* gene were retrieved using the NCBI Genome Data Viewer [23]. Characteristics of silencers: silencer a: 283 bp (7 CpGs); silencer b: 441 bp (54 CpGs); silencer c: 161 bp (23 CpGs); silencer d: 199 bp (3 CpGs); silencer e: 296 bp (3 CpGs); silencer f: 441 bp (63 CpGs); silencer g: 201 bp (15 CpGs); silencer h: 91 bp (3 CpGs). Genomic positions (GRCh38.p14): NC_000010.11: 128,078,544–128,078,839 (silencer a), 128,149,999–128,150,439 (silencer b), 128,210,528–128,210,688 (silencer c), 128,626,385–128,626,583 (silencer d), 129,446,224–129,446,519 (silencer e), 129,466,880–129,467,320 (silencer f), 129,467,340–129,467,540 (silencer g), 129,604,668–129,604,758 (silencer h)

Silencer regions were selected based on annotations from the NCBI Genome Data Viewer (GRCh38.p14; NC_000010.11) (Fig. 1). Regions within and surrounding the *MGMT* locus, including distal regions located up to approximately 1.5 Mb upstream of *MGMT* were included for analysis, without prior functional validation of *MGMT*-specific regulatory activity. DNA sequences of the respective silencer regions were extracted based on the indicated genomic positions and used for primer design with PyroMark Assay Design Software 2.0.1.15 (Qiagen, Hilden, Germany).

Primers were designed to cover intergenic CpG sites (Sa-04–Sa-07: “silencer a”; Sb-30–Sb-43: “silencer b”; Sc-01–Sc-08 and Sc-19–Sc-23: “silencer c”; Sd-01, Sd-02: “silencer d”; Se-02, Se-03: “silencer e”); promoter-associated CpG sites (PS-33–PS-38: “silencer f“ and PS-72–PS-83; PS-91–PS-94: “silencer g”), as well as intron-associated CpG sites (Sg-12–Sg-15 + 1: “silencer g”; Sh-01, Sh-02: “silencer h”) (Fig. 1A, B). Primer sequences are listed in Supplementary Table S3.

Assays were optimized, applying different annealing and elongation temperatures, as well as primer concentrations for PCR (run on QuantStudio 5 thermal cycler (Thermo Scientific, Waltham, MA, USA)), using bisulfite converted human DNA standards (Zymo Research, Irvine, CA, USA). For PCR amplification the PyroMark PCR Kit (Qiagen, Hilden, Germany) was used, including 1x PyroMark MasterMix, 1x CoralLoad concentrate, forward and reverse primer, EvaGreen HRM dye as well as 2.5 ng bisulfite converted DNA. In each PCR experiment two no-template controls were run, replacing DNA with nuclease-free water.

Ten µL of amplicons were sequenced on the PyroMarkQ48 Autoprep instrument, using PyroMark Q48 Advanced CpG Reagents, PyroMark Q48 Autoprep Accessories, and PyroMark Q48 Autoprep software 2.4.2, according to the manufacturer’s protocol. Each sample was analyzed in two individual PCR/PSQ runs. Bisulfite conversion controls included in the pyrosequencing assays confirmed efficient bisulfite conversion.

### Data analysis and statistics

PCR amplification curves were acquired using QuantStudio Design & Analysis Software v1.4.3 (ThermoScientific, Waltham, MA, USA). For evaluation of pyrograms and export of methylation data, PyroMark Q48 Autoprep software 2.4.2 was used. R software (version 4.3.1) served for statistical analysis and graphical presentation of results. Assessed R-packages are listed in Supplementary Information.

Mann–Whitney U tests were applied to assess differences between two groups. Spearman correlation coefficients were used to assess associations between variables. For correlation analyses, pairwise complete observations were used.

All *p*-values were adjusted for multiple testing using the Benjamini–Hochberg (BH) procedure, and only adjusted *p*-values were considered for statistcal inference. A detailed overview of sample sizes and data availability across all analyses is provided in Supplementary Table S4.

## Results

### Methylation levels of silencer-annotated regions

**Fig. 2:**
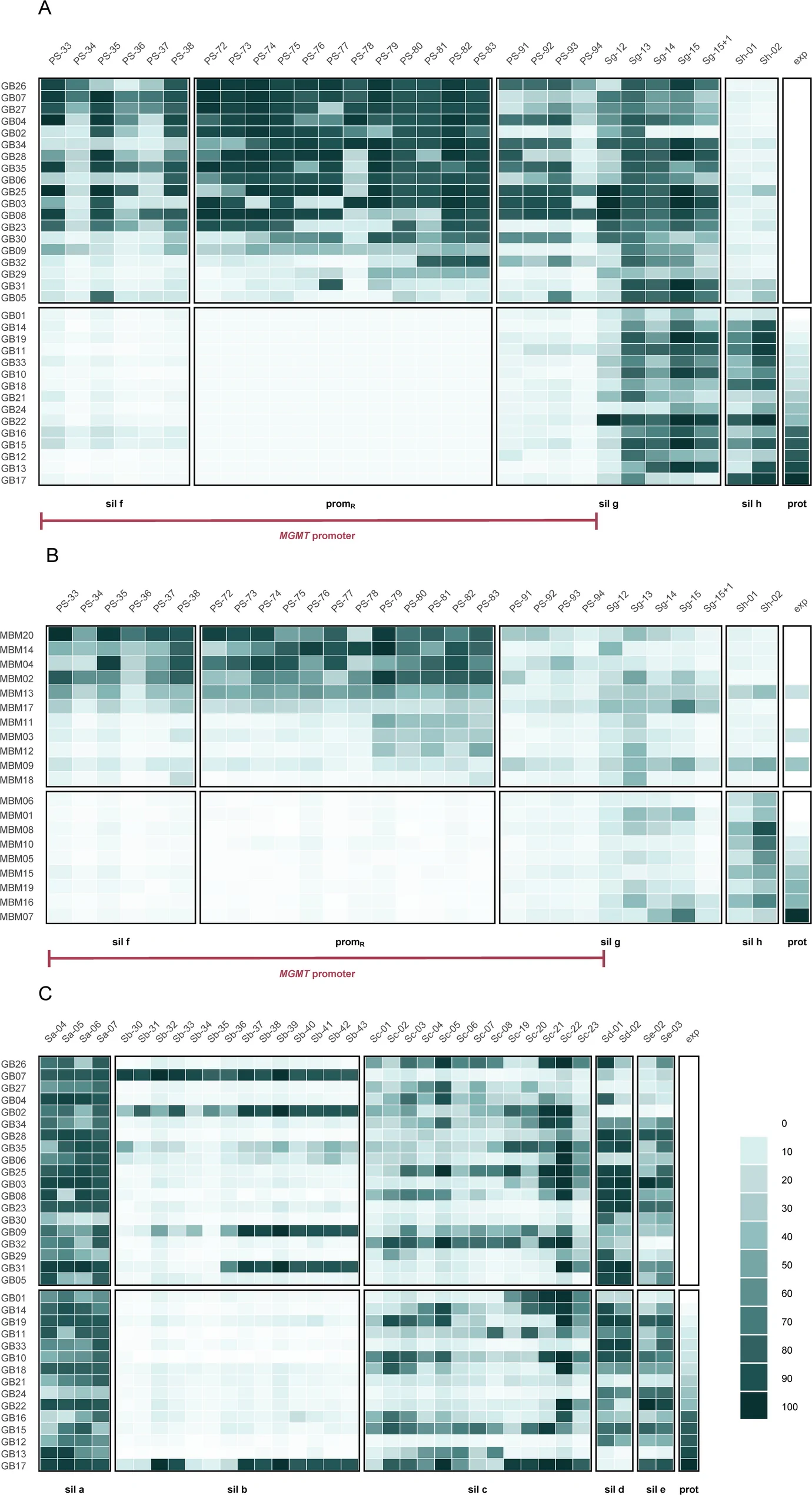
Heatmap displaying DNA methylation levels of CpGs within silencer-associated regions in GB and MBM samples. **A**, **B** Promoter- and intron-associated silencers. **A** GB samples. **B** MBM samples. prom_R_ refers to CpGs 72–83 located in the *MGMT* promoter region commonly used for biomarker analysis. **C** Intergenic silencers (GB samples). Upper panel: samples with a methylated MGMT promoter (defined as at least one CpG site with a methylation level > 8%). Lower panel: samples with an unmethylated *MGMT* promoter (all CpG sites showing a methylation level ≤ 8%). Within each panel, samples are arranged according to MGMT protein expression levels, as assessed by Western blot analysis. PCR and PSQ were performed in two independent runs. Color scale: 0% (white) to 100% (dark green). PS: CpG sites in promoter-associated silencers; Sa–Se, Sg, Sh: CpG sites in silencers a–e, g and h, numbered according to their position within the respective silencer; exp: expression; prot: protein

In this study, we analyzed DNA methylation at 52 CpG sites across eight silencer-annotated regions (“silencers a–h”) located within or near MGMT, including one CpG site directly downstream of silencer g. Figures 2A and B show DNA methylation levels of promoter-associated (PS) and intron-associated silencers in GB and MBM samples, respectively. Specifically, CpGs PS-33–PS-38 and PS-72–PS-83 are located within silencer f, while CpGs PS-91–PS-94 are located within silencer g. The remaining CpGs of silencer g are located in MGMT intron 1, whereas silencer h is located in intron 2. Methylation levels of CpGs PS-72–PS-83, which are commonly used as a predictive biomarker in GB, were determined previously [16, 19]. Overall, the heatmaps revealed comparable methylation patterns across promoter-associated CpG sites in GB and MBM, with a tendency toward higher methylation levels in GB.

Methylation of silencers a–e was analyzed in GB, providing additional insights into intergenic silencer elements upstream of the *MGMT* gene (Fig. 2C). Silencer e is annotated as both a silencer and an enhancer. The heatmap reveals distinct methylation patterns across individual silencer elements, with substantial variability between tumors. Due to limited material availability in MBM samples, intergenic silencer methylation was not analyzed in this tumor entity.

To enable comparison across different genomic contexts, CpGs were divided into the following groups: CpGs located in intergenic silencers; CpGs in the *MGMT* promoter region commonly used as biomarker in GB (prom_R_); CpGs located upstream or downstream of prom_R_ within the promoter; and CpGs located in intronic silencers.

**Fig. 3:**
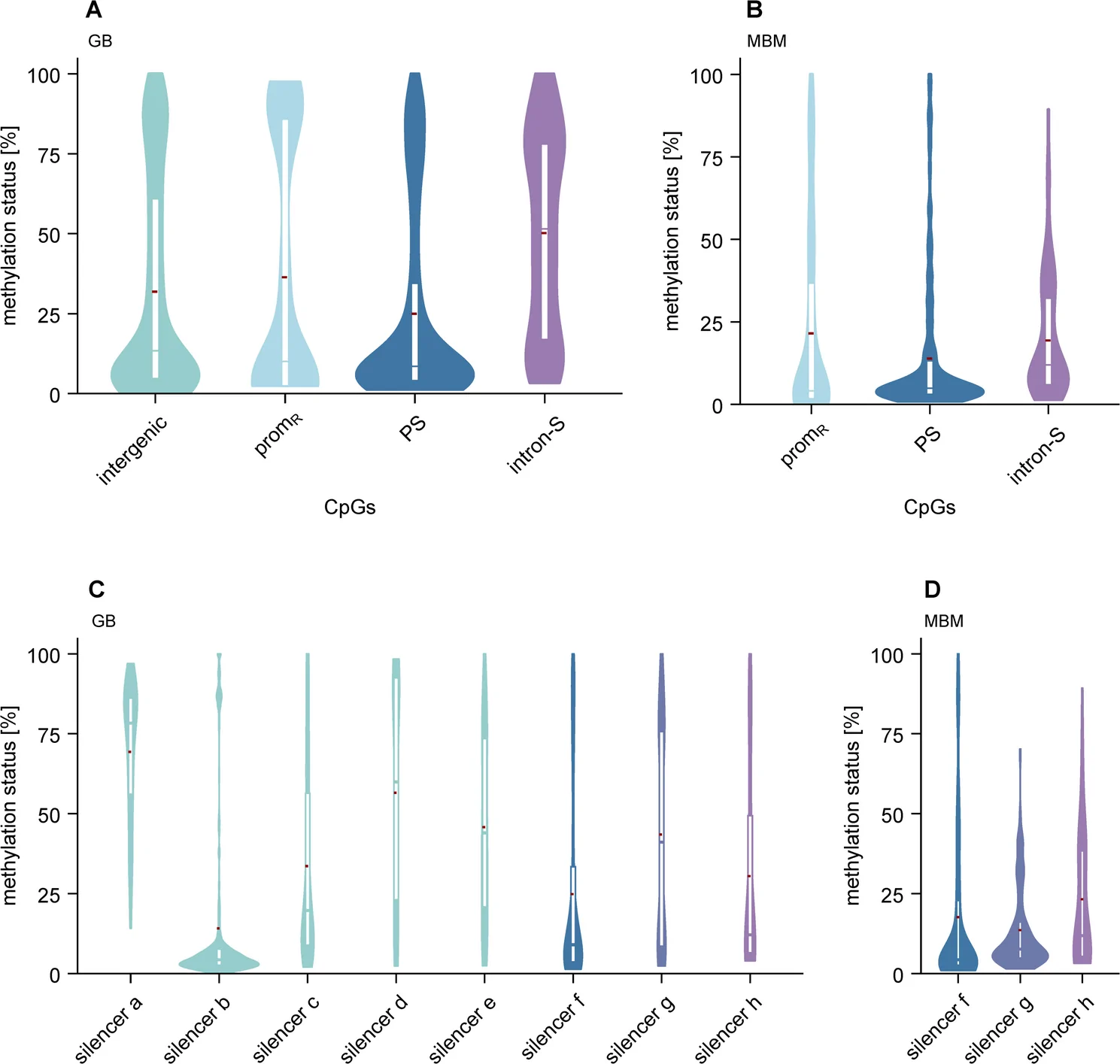
Distinct methylation distribution of silencer-annotated regions in GB and MBM samples. **A**,** C** GB samples, **B**,** D** MBM samples. **A**,** B** CpGs were grouped into intergenic silencers; CpGs within prom_R_; CpGs in the promoter upstream or downstream of prom_R_ (PS, comprising CpGs PS-33─PS-38 and PS-91–PS-94); and intronic silencers (intron-S). **C**,** D** Methylation distribution of individual silencers

Methylation patterns of silencer groups revealed distinct differences between GB and MBM (Fig. 3). In GB, prom_R_ (comprising PS-72–PS-83) was unmethylated in many samples, a pattern also observed for CpGs immediately upstream or downstream of prom_R_ (Fig. 3A). In the remaining GB samples, prom_R_ tended to show higher methylation levels than the promoter-associated CpGs outside of prom_R_. At prom_R_ CpG sites, median methylation levels ranged from 3.2% to 28.2%, compared to 5.7%–15.3% and 4.4%–11.6% for CpGs upstream and downstream of prom_R_, respectively.

In MBM, some samples had unmethylated prom_R_ CpGs, while methylated samples displayed heterogeneous methylation levels (Fig. 3B). CpGs outside prom_R_ within the promoter were generally less methylated than those within prom_R_. Across prom_R_ CpG sites, median methylation levels ranged from 2.4% to 11.9%, compared to 2.9%–6.1% and 4.1%–6.9% for CpGs upstream and downstream of prom_R_, respectively.

Analysis of intronic silencers revealed that in GB samples, the degree of methylation varied widely (Fig. 3C). In MBM, intronic silencer CpGs ranged from low to intermediate methylation (median levels of CpG sites ranging from 3.9% to 31.0%, Fig. 3D).

Intergenic silencers, analyzed only in GB, exhibited a broad spectrum of methylation (Fig. 3C). At most CpG sites, silencer b showed low methylation across all samples (median methylation ≤ 7.7%), whereas silencer a showed relatively high median methylation levels across all samples, ranging from 70.6% to 80.6%. In silencers c–e, GB samples displayed heterogeneous methylation levels.

**Fig. 4:**
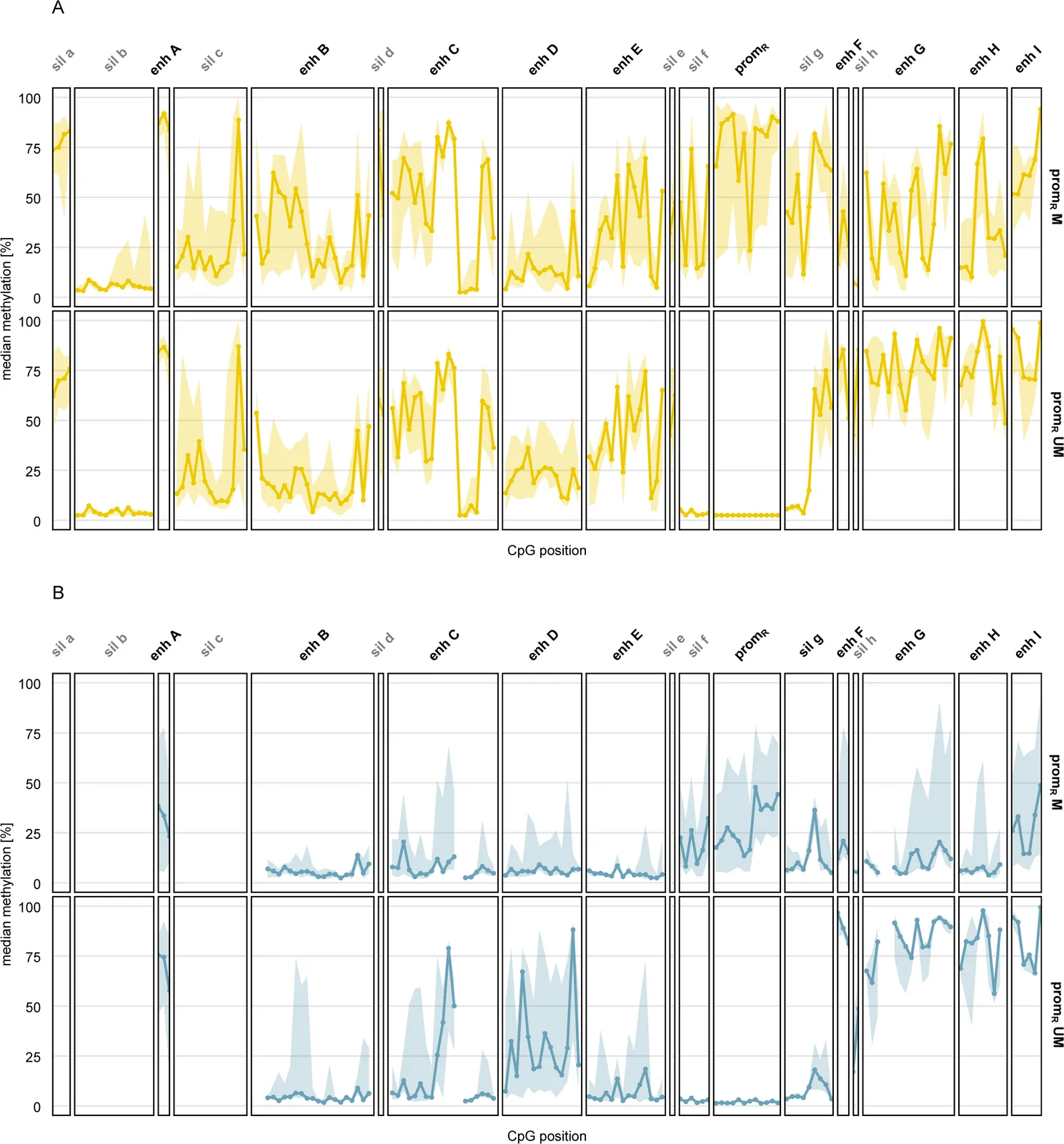
Distinct CpG methylation patterns across *MGMT*-associated regulatory elements in GB and MBM samples. **A**,** B** Median DNA methylation levels (%) across the regulatory landscape of *MGMT*. **A** GB, **B** MBM. CpG sites are arranged according to their genomic order, spanning promoter, enhancer, and silencer-annotated elements. Lines represent median methylation levels across samples, and shaded areas indicate the standard error of the mean. Upper panel: prom_R_ methylated samples, lower panel: prom_R_ unmethylated samples. Note that not all CpG sites within each regulatory element were analyzed; gaps between CpGs may occur (see Fig. 1 for the exact positions of the analyzed CpGs)

### DNA methylation landscape of MGMT regulatory elements

To obtain a comprehensive view of the DNA methylation landscape of *MGMT* regulatory elements in GB and MBM, median DNA methylation levels were plotted across promoter, enhancer, and silencer-annotated elements according to their genomic order (Fig. 4). Figure 4A shows data from GB and Fig. 4B from MBM. Due to the low CpG density in enhancers and silencers, multiple assays were required, and data were not obtained for all CpG sites within each element (see Fig. 1 for the exact positions of the analyzed CpGs).

Within almost all regulatory elements, methylation levels varied substantially between CpG sites, resulting in a characteristic zigzag-like pattern across the analyzed regions. Shaded areas indicate variability in methylation levels across the sample sets. Across prom_R_ CpG sites, PS-78 showed markedly lower median methylation than surrounding CpGs in GB samples (Fig. 4A). The zigzag-like pattern was particularly pronounced in several intergenic elements, including silencer c and enhancers C and E in both promoter-unmethylated and promoter-methylated GB samples. For instance, median methylation levels of CpG sites Sc-21 to Sc-23 were 38.4%, 88.7%, and 21.5% in promoter-methylated samples and 15.6%, 87.0%, and 35.5% in promoter-unmethylated samples, illustrating the pronounced CpG-specific differences. In enhancer E, the zigzag-like pattern originated mainly from differences between adjacent CpGs, as only two CpGs (CpGs 18 and 19) were not covered between the analyzed sites. In enhancer C, a marked increase in median methylation was observed from ~ 30% at CpG 14 to ~ 80% at CpG 15 in both promoter-methylated and -unmethylated GB samples. Additional pronounced changes within enhancer C occurred between non-adjacent CpGs: between CpG 18 and CpG 24, methylation decreased from ~ 80% to almost unmethylated levels, whereas between CpG 27 and CpG 37, methylation increased to ~ 62%. In MBM samples, zigzag-like patterns resulting from differences between adjacent CpGs were mainly observed in enhancer D and in promoter-unmethylated samples.

With few exceptions, the overall methylation landscape differed between GB and MBM with respect to both absolute methylation levels and CpG-specific patterns, suggesting tumor-type–specific variation. Notably, the methylation landscape of intronic silencer h and enhancers G–I was relatively similar in GB and MBM, but only in promoter-unmethylated samples. For silencer g, a marked increase in methylation was observed in GB samples from the last promoter CpG (PS-94, corresponding to CpG 07 in silencer g; Fig. 1B) to CpG 12 located in intron 1 of the *MGMT* gene. This increase at the promoter-intron 1 transition was observed in both promoter-methylated and promoter-unmethylated samples. In promoter-unmethylated samples, however, it marked a transition from unmethylated promoter CpGs to higher methylation levels within intron 1.

### Association between DNA methylation in silencer-annotated regions, promoter, and enhancer regions

**Fig. 5:**
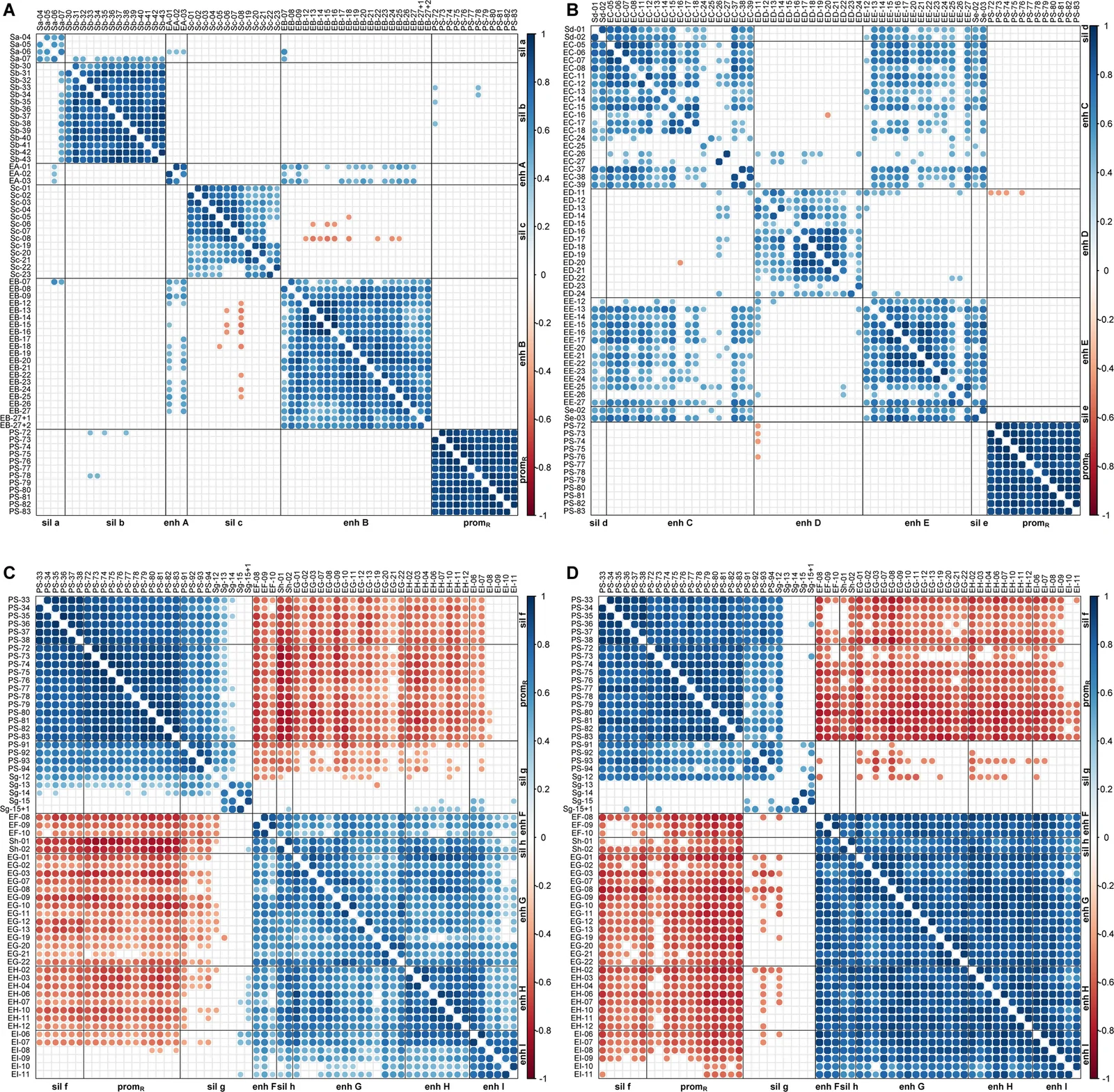
Significant Spearman correlation coefficients for relationships between DNA methylation levels of individual CpGs. **A** silencers a–c and enhancers A and B, **B** silencers d and e and enhancers C–E, **C** silencers f–h and enhancers F–I. Color code: dark red (−1) to dark blue (+ 1). Methylation levels of CpG sites PS-72–83 and CpGs in enhancers were acquired previously [16, 17]. Correlation coefficients were assessed using pairwise complete comparison and Benjamini-Hochberg (BH) adjustment for multiple testing. Sil: silencer, enh: enhancer

Correlation analysis was performed to examine associations between CpG sites in the *MGMT* promoter, intronic silencers, and enhancer regions in GB (Fig. 5A–C) and MBM (Fig. 5D) samples.

Strong positive correlations were observed among CpGs within prom_R_ and the promoter-associated CpGs upstream and downstream of prom_R_ in both GB (Fig. 5C) and MBM (Fig. 5D). In contrast, while CpGs located in *MGMT* intron 1 showed more variable correlations, the two CpGs in intron 2 were strongly positively correlated with enhancers located within intron 2 or intron 3, indicating region-specific co-methylation.

In MBM (Fig. 5D), correlation patterns of prom_R_ and upstream promoter-associated CpGs resembled those in GB, but correlations among downstream promoter-associated CpGs were less pronounced. Among the intergenic silencers, CpGs in silencers d and e formed distinct correlated clusters (Fig. 5B), whereas almost no correlations were found among silencers a–c (Fig. 5A).

### Association of DNA methylation in silencer-annotated regions with MGMT protein expression

**Fig. 6:**
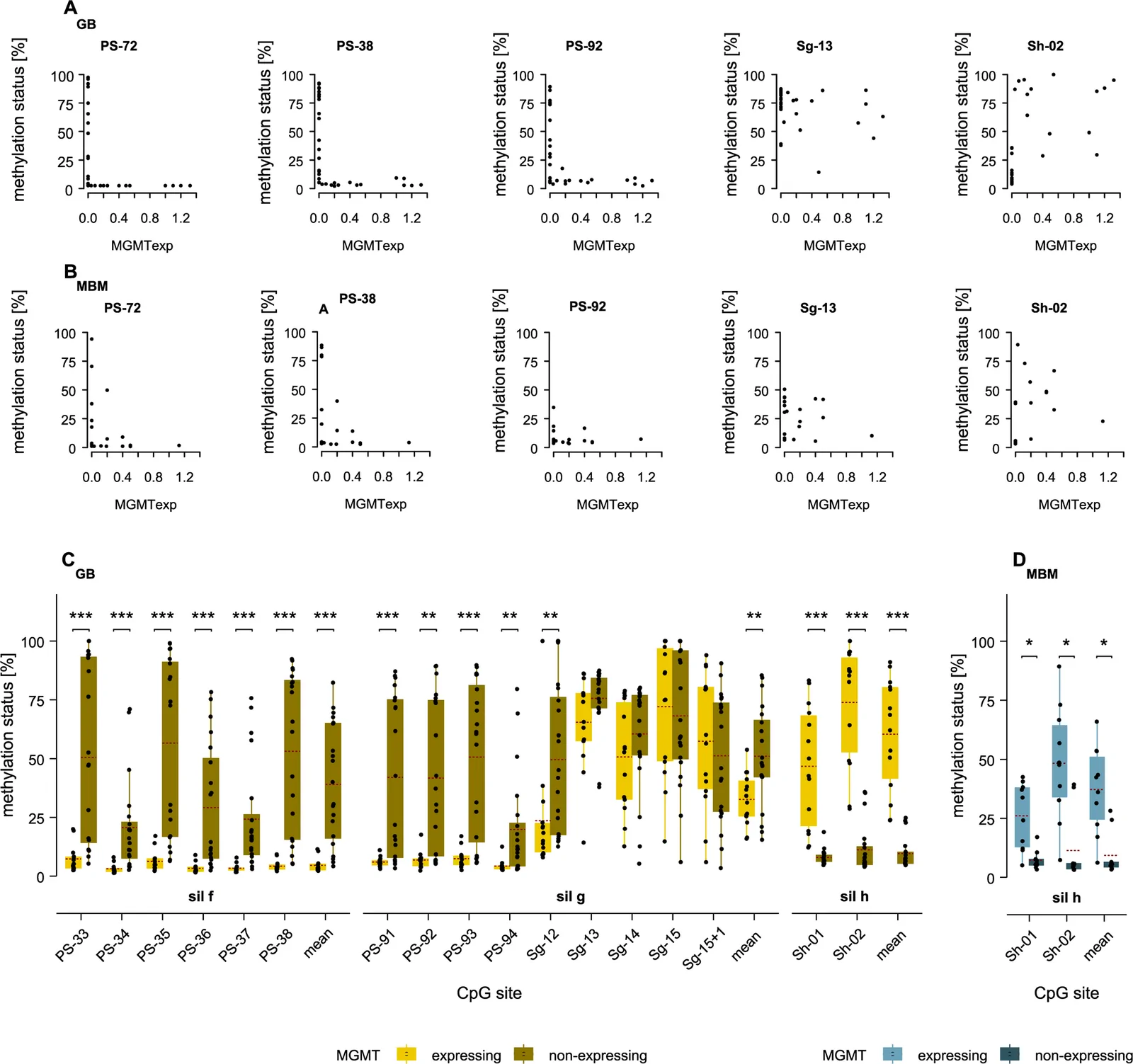
Relationship between methylation of silencer-annotated regions and MGMT protein expression in GB and MBM samples. **A**,** B** Scatter plots illustrating the association between methylation levels at selected promoter-associated and intronic silencer CpGs (PS-72, PS-38, PS-92, Sg-13, and Sh-02) and MGMT protein expression in **A** GB and **B** MBM. **C**,** D** Boxplots showing differences in CpG methylation between MGMT protein-expressing and non-expressing samples in **C** GB and **D** MBM. The mean methylation level is indicated by a red dotted line. Statistical significance was assessed using the Mann-Whitney U test (*p < 0.05, **p < 0.01, ***p < 0.001)

To explore the functional consequences of methylation at promoter-associated and intronic silencer CpGs, we examined the relationship between CpG methylation levels and MGMT protein expression in GB and MBM samples. In GB, an L-shaped relationship was observed between MGMT protein expression and methylation at prom_R_ CpGs (PS-72–PS-83) as well as promoter-associated CpGs located upstream (PS-33–PS-38) and downstream (PS-91–PS-94) of prom_R_ (Fig. 6A). In contrast, no clear association with MGMT protein expression was observed for CpGs Sg-12–Sg-15 + 1 located in intron 1 or for CpGs Sh-01 and Sh-02 located in intron 2.

When the samples were stratified into MGMT protein-expressing and non-expressing groups, significantly higher methylation levels were observed in the non-expressing group at CpGs PS-33–PS-38 and their mean methylation (*p* < 0.01), as well as at CpGs PS-91–PS-94, Sg-12, and silencer g mean methylation (*p* ≤ 0.007) (Fig. 6C). In contrast, CpGs Sh-01 and Sh-02, as well as their mean methylation, showed significantly higher methylation levels in MGMT protein-expressing samples (*p* < 0.001).

Consistent with the findings in GB, an L-shaped relationship between promoter-associated CpG methylation and MGMT protein expression was also observed in MBM samples, although some samples deviated from this pattern (Fig. 6B). In contrast to promoter-associated CpGs, methylation levels of intronic silencer CpGs did not show an L-shaped relationship with MGMT protein expression. However, methylation at CpGs Sh-01 and Sh-02, as well as their mean methylation, was significantly higher in MGMT-expressing than in non-expressing MBM samples (*p* < 0.05) (Fig. 6D).

Scatter plots illustrating the relationship between CpG methylation and MGMT protein expression for all analyzed promoter-, enhancer-, and silencer-associated CpGs are provided in Supplementary Fig. 1.

### Association of DNA methylation in silencer-annotated regions with overall survival

**Fig. 7:**
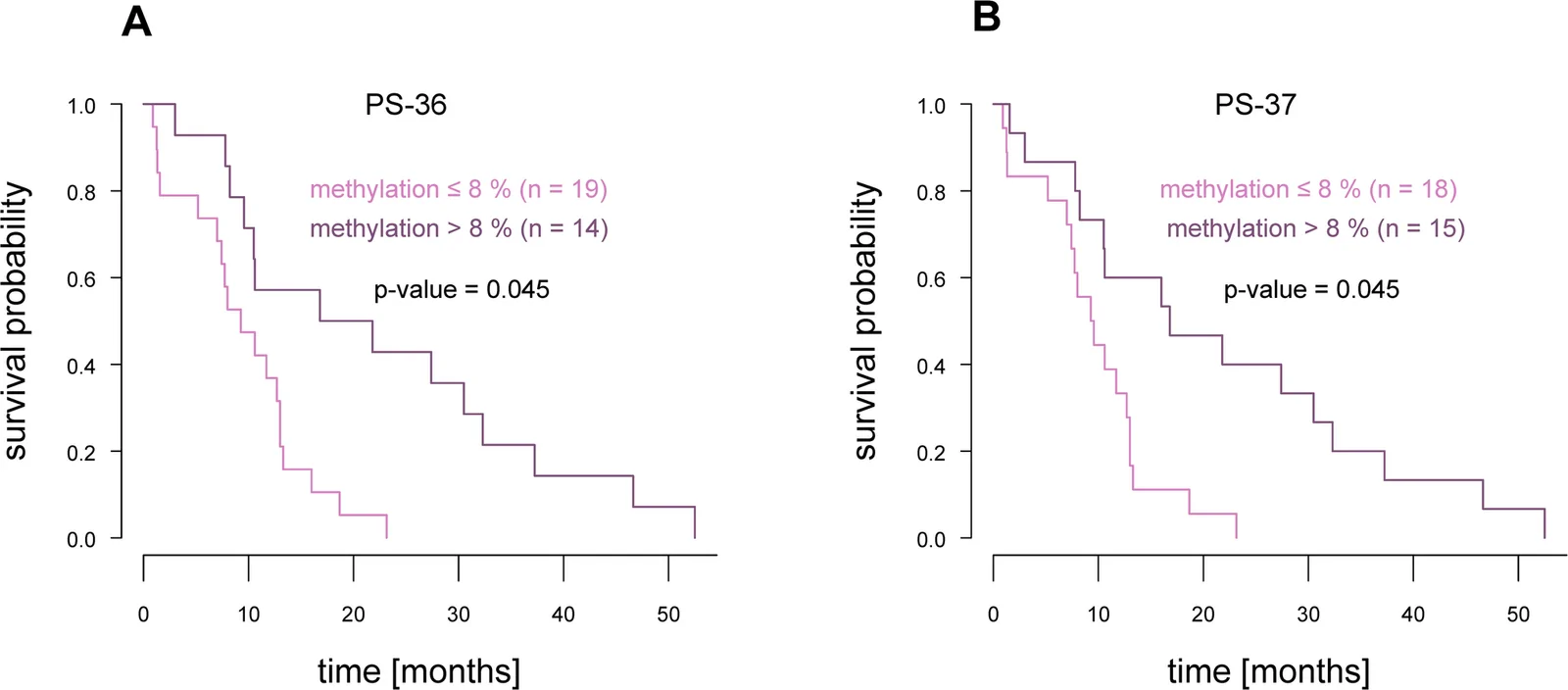
Representative Kaplan-Meier survival curves indicating the association of methylation levels in silencer-annotated regions with OS in GB. Kaplan-Meier survival curves for GB samples stratified by methylation (≤ 8% or > 8%) at **A** PS-36 and **B** PS-37. *p* values were determined by log-rank test

In GB, CpGs PS-36 and PS-37 in promoter-associated silencer f showed prognostic potential for overall survival. Patients with methylation levels above 8% at these CpGs, a commonly used clinically relevant cutoff for *MGMT* promoter methylation [18], exhibited significantly longer overall survival compared with patients showing methylation levels ≤ 8% (Fig. 7A, B).

In MBM, overall survival was not associated with DNA methylation in silencer-annotated regions.

#### Association of silencer methylation with age and sex

We also explored whether silencer methylation is associated with patient age and sex in GB and MBM. No robust association between DNA methylation and patient age and sex was observed after accounting for multiple testing.

## Discussion

Precise spatiotemporal regulation of gene expression requires a complex interplay of regulatory elements, including promoters, enhancers, and silencers [24, 25]. Although silencers have been identified across the human genome [26–30], they have received considerably less attention than promoters or enhancers, and their regulation and functional contribution to gene expression remain incompletely understood [31, 32]. Silencers function as negative regulatory elements that repress gene transcription [33]. However, increasing evidence suggests that many regulatory elements are context dependent, and silencers can act also as enhancers depending on the tissue type or cellular environment [29, 34, 35]. Recent studies further indicate that silencers may be regulated epigenetically by DNA methylation [27, 36] and that aberrant silencer methylation is associated with various diseases, including cancer [36].

To gain insight into the role of silencer DNA methylation in cancer, we analyzed CpG methylation levels within silencers in two brain tumors, GB and MBM. In GB, *MGMT* promoter methylation has been extensively studied and is widely used as a predictive biomarker [6], whereas its relevance in MBM remains controversially discussed [37, 38]. Although both tumor entities occur in the brain and share a similar microenvironment, primary and secondary brain tumors differ substantially in their evolutionary history and are therefore likely exposed to distinct microenvironmental cues [39]. By generating a DNA methylation landscape of *MGMT* regulatory elements, combining silencer methylation data obtained in the present study with previously published methylation data for the promoter and enhancers [16, 17, 19], we aimed to obtain deeper insight into *MGMT* regulation in these two tumor entities.

We focused on regulatory regions annotated as silencers in the NCBI Genome Data Viewer. The analyzed silencer elements were defined based on genomic annotation and their direct regulatory interaction with *MGMT* has not been experimentally validated. Our study included CpG sites spanning approximately 1.5 Mb upstream of *MGMT*. Intergenic silencer e has also been described as an enhancer. A particular challenge during assay development was the relatively low CpG density within silencer regions. Consequently, ten independent assays were established to cover a total of 52 CpG sites, including 35 CpGs in intergenic silencers upstream of the *MGMT* gene, ten promoter-associated CpGs located upstream and downstream of prom_R_ (the promoter region most commonly analyzed [40–42]), and seven CpG sites within intronic silencers.

Our data reveal that the degree of silencer methylation strongly depends on the genomic location of the respective silencer. Moreover, both shared and tumor-type-specific methylation patterns were observed in GB and MBM, consistent with our previous findings for *MGMT* enhancers [19]. Differences between the two tumor entities were mainly reflected in absolute methylation levels, with a tendency toward higher silencer methylation in GB. In promoter-methylated GB and MBM samples, CpGs within prom_R_ tended to show higher methylation levels than the promoter-associated CpGs outside prom_R_. In GB, intronic silencer methylation varied widely, whereas in MBM, intronic CpGs generally showed low to intermediate methylation levels. Intergenic silencers, which were analyzed only in GB, displayed a broad spectrum of methylation levels.

A comprehensive assessment of methylation data obtained in this study together with previously published data on *MGMT* promoter and enhancer methylation [16, 17, 19] revealed distinct methylation landscapes across *MGMT* regulatory elements. Pronounced CpG-to-CpG variability in DNA methylation resulted in characteristic zigzag-like methylation patterns across several regulatory elements. A striking feature was a pronounced increase in methylation at the transition from the *MGMT* promoter to intron 1, suggesting that regulatory regions within the *MGMT* locus may be epigenetically organized into distinct domains. Despite these pronounced differences in absolute methylation levels between neighboring CpG sites, correlation analyses demonstrated that CpG methylation within the *MGMT* promoter and its adjacent regions is highly coordinated across samples in both GB and MBM, whereas correlations with CpGs located in more distal regulatory elements were more variable. Notably, CpGs located within intron 2 showed coordinated methylation with enhancers located in the same genomic region.

A limitation of this study is the incomplete coverage of intergenic silencer-annotated regions in MBM due to limited sample material. Consequently, the *MGMT* regulatory landscape in MBM is restricted to promoter- and intronic regions, whereas GBM allows a more comprehensive analysis including intergenic regions. This asymmetry should be considered when interpreting cross-tumor comparisons. However, our findings indicate the coexistence of strong site-specific methylation differences and region-specific co-methylation patterns, highlighting the complex regulatory architecture of the *MGMT* locus [16, 17, 19]. DNA methylation at regulatory elements may influence gene expression by modulating the binding of transcriptional repressors and other regulatory proteins. In this context, gene expression is increasingly recognized to be controlled by networks of regulatory elements rather than by individual promoters alone [43]. Our findings therefore support the concept that coordinated epigenetic changes across multiple regulatory regions may contribute to *MGMT* regulation in brain tumors [36].

An L-shaped relationship between DNA methylation of CpGs located within prom_R_ and MGMT protein expression has previously been reported in GB and MBM [17, 19]. In the present study, a similar L-shaped relationship was also observed for promoter-associated CpGs located upstream and downstream of prom_R_ in both tumor entities, although some MBM samples deviated from this pattern. In contrast, an L-shaped relationship with MGMT protein expression was not observed for CpGs located within intron 1 or intron 2 in either GB or MBM.

However, the two analyzed CpGs within intronic silencer h (intron 2) exhibited significantly higher methylation levels in MGMT-expressing samples than in non-expressing samples in both tumor entities. Intronic regulatory elements, particularly those located in early introns, have been suggested to play important roles in the modulation of gene transcription [44]. DNA methylation at silencer h may therefore be associated with altered regulatory activity at this locus and could contribute to MGMT expression differences.

In GB, methylation of promoter-associated CpGs upstream of prom_R_ showed prognostic potential for overall survival. Associations of silencer methylation with patient age and sex were absent in both GB and MBM samples.

## Conclusions

Our study demonstrates that DNA methylation across *MGMT* regulatory elements is highly CpG-specific and extends beyond the classical promoter region to enhancer- and silencer-annotated regions. Promoter-associated and selected intronic CpGs show associations with MGMT protein expression and, in some cases, overall survival. These results highlight pronounced epigenetic heterogeneity across the *MGMT* locus and emphasize the importance of CpG-resolved analyses for biomarker development and future functional studies.

## Supplementary Information

Below is the link to the electronic supplementary material.

Supplementary Material 1

## Data Availability

All data supporting the findings of this study are available within the paper and its Supplementary Information.

## Abbreviations

CpG: CpG dinucleotide
GBM: Glioblastoma
MBM: Melanoma brain metastases
MGMT: O6-methylguanine-DNA methyltransferase
OS: Overall survival
PCR: Polymerase chain reaction
PSQ: Pyrosequencing
TMZ: Temozolomide
