# Supplementary material for: DNA methylation of silencer-annotated regions at the MGMT locus in the context of promoter and enhancer methylation in glioblastoma and melanoma brain metastases
Source: Clin Epigenetics. 2026 Aug 1;18:198. doi: 10.1186/s13148-026-02204-y (PMC13721255; doi:10.1186/s13148-026-02204-y)
Supplement: Supplementary file 1 [file 13148_2026_2204_MOESM1_ESM.docx]

*Supplementary Information*

DNA methylation of silencer-annotated regions at the *MGMT* locus in the context of promoter and enhancer methylation in glioblastoma and melanoma brain metastases

Katharina Pühringer ^1,2^, Fabian Firlinger ^1^, Benno Fehringer ^1^, Serge Weis ^3,5^, Sabine Spiegl-Kreinecker ^4,5^, and Margit Cichna-Markl ^1,^*

1 Department of Analytical Chemistry, Faculty of Chemistry, University of Vienna, Vienna, Austria

2	Vienna Doctoral School in Chemistry (DoSChem), University of Vienna, 1090 Vienna, Austria

3	Division of Neuropathology, Department of Pathology and Molecular Pathology, Kepler University Hospital GmbH, Johannes Kepler University, Linz, Austria

4	Department of Neurosurgery, Johannes Kepler University, Linz, Kepler University Hospital GmbH, Austria

5	Clinical Research Institute for Neurosciences, Johannes Kepler University, Linz, Austria

*****	Correspondence: [margit.cichna@univie.ac.at](mailto:margit.cichna@univie.ac.at)

*Supplementary Tables*

**Table S1.** GB patient characteristics

| **patient** | **age [y]** | **sex** | **MGMT protein expression** | **OS [m]** | **primary therapy** | **adjuvant therapy** |
| --- | --- | --- | --- | --- | --- | --- |
| GB01 | 64 | female | 0.00 | 7.43 | R-Ch-T | none |
| GB02 | 85 | male | 0.00 | 3.00 | none | none |
| GB03 | 53 | female | 0.00 | 52.50 | R-Ch-T | none |
| GB04 | 67 | female | 0.00 | 46.63 | RT | none |
| GB05 | 57 | female | 0.00 | 10.50 | RT | CCNU |
| GB06 | 46 | male | 0.00 | 30.50 | R-Ch-T | TMZ + CCNU |
| GB07 | 50 | female | 0.00 | 27.40 | RT | TMZ |
| GB08 | 74 | female | 0.00 | 7.79 | R-Ch-T^1^ | Avastin + TMZ |
| GB09 | 48 | male | 0.00 | 1.55 | none | none |
| GB10 | 64 | male | 0.20 | 11.70 | R-Ch-T | TMZ |
| GB11 | 73 | female | 0.16 | 10.60 | R-Ch-T | no |
| GB12 | 44 | male | 1.10 | 13.00 | R-Ch-T | TMZ |
| GB13 | 65 | male | 1.20 | 9.27 | RT^1^ | none |
| GB14 | 69 | male | 0.04 | 8.00 | RT | TMZ + CCNU |
| GB15 | 73 | female | 1.10 | 7.00 | none | none |
| GB16 | 83 | male | 1.00 | 9.57 | RT | none |
| GB17 | 74 | male | 1.32 | 5.19 | R-Ch-T | none |
| GB18 | 44 | male | 0.25 | 23.15 | R-Ch-T | TMZ |
| GB19 | 48 | male | 0.09 | 18.67 | R-Ch-T | TMZ |
| GB21 | 60 | male | 0.40 | 13.00 | R-Ch-T | Avastin |
| GB22 | 53 | male | 0.54 | 0.89 | none | none |
| GB23 | 47 | female | 0.00 | 37.25 | R-Ch-T | TMZ |
| GB24 | 64 | male | 0.49 | 7.73 | R-Ch-T | TMZ |
| GB25 | 67 | female | 0.00 | 16.80 | R-Ch-T | TMZ |
| GB26 | 75 | female | 0.00 | 8.22 | RT | none |
| GB27 | 52 | female | 0.00 | n.a. | R-Ch-T | TMZ |
| GB28 | 63 | male | 0.00 | 21.80 | R-Ch-T | TMZ |
| GB29 | 79 | female | 0.00 | 1.31 | none | none |
| GB30 | 58 | male | 0.00 | 16.00 | R-Ch-T | TMZ |
| GB31 | 58 | female | 0.00 | 13.30 | R-Ch-T | TMZ |
| GB32 | 71 | male | 0.00 | 1.25 | none | none |
| GB33 | 57 | male | 0.20 | 12.70 | R-Ch-T | TMZ |
| GB34 | 53 | male | 0.00 | 32.30 | R-Ch-T | TMZ |
| GB35 | 64 | male | 0.00 | 10.60 | R-Ch-T | none |

**Table S2.** MBM patient characteristics

| **sample** | **age**  **[y]** | **sex** | **MGMT protein expression** | **MGMT mRNA expression** | **OS [m]** |
| --- | --- | --- | --- | --- | --- |
| MBM01 | 39 | male | 0.00 | 1.96 | 6.08 |
| MBM02 | 71 | female | 0.00 | 0.00 | 2.76 |
| MBM03* | 62 | female | 0.20 | 0.20 | 26.66 |
| MBM04 | 76 | male | 0.00 | 1.06 | 1.08 |
| MBM05 | 80 | male | 0.19 | 0.38 | 2.5 |
| MBM06 | 61 | male | 0.00 | 3.86 | 2.24 |
| MBM07 | 75 | male | 1.13 | 0.66 | 5.52 |
| MBM08 | 49 | male | 0.03 | 1.03 | 2.43 |
| MBM09 | 62 | male | 0.40 | 1.50 | 2.47 |
| MBM10 | 48 | male | 0.12 | 1.80 | 6.21 |
| MBM11* | 70 | female | 0.00 | 0.15 | 2.89 |
| MBM12** |  |  | 0.00 | 0.22 |  |
| MBM13 | 38 | female | 0.20 | 0.90 | 5.49 |
| MBM14 | 59 | female | 0.00 | 1.52 | 0.1 |
| MBM15 | 40 | male | 0.40 | 1.70 | 2.89 |
| MBM16 | 44 | female | 0.50 | 3.50 | 13.74 |
| MBM17 | 65 | female | 0.00 | 0.09 | 5.23 |
| MBM18 | 41 | male | 0.00 | 0.00 | 5.59 |
| MBM19 | 61 | female | 0.50 | 1.00 | n.a. |
| MBM20* | 50 | female | 0.00 | 0.70 | 170.97 |

*first recurrence; **second recurrence; wt: wildtype; y: years. MBM11 and MBM12 originated from the same patient. n.a.: not available.

**Table S3**. Silencer coordinates, primer sequences, amplicon length and analyzed CpG sites.

| **silencer** | **coordinates** | **primer set** | **primer sequence (5´→3´)** | **length [bp]** | **CpGs  analyzed** |
| --- | --- | --- | --- | --- | --- |
| **a**  7 CpGs | NC_000010.11: 128078544–128078839 | A | F: GGTATTGGAGATGTAGAGGTGAAT  R: [Btn] CACATATAAACTTATTCCAAAACTCCTAT S: AGATTTTATTTGTG | 102 | 04–07 |
| **b**  54 CpGs | NC_000010.11: 128149999–128150439 | A | F: GGAGGGGAAGGGATTTTATT  R: [Btn] ATTCCCAAACTCCAAATAACCA  S: ATTTTATTTTGTATTTTTTATA | 130 | 30–43 |
| **c**  23 CpGs | NC_000010.11: 128210528–128210688 | A | F: GGGGTGTTYGTAGAGAAG  R: [Btn] ATTCCRTTTCTCAAACACAC  S: GTTTAATTTTYGGAGGGATTGTT | 143 | 01–08 |
|  |  | B | F: GAGTATTTYGGGGTTTTTAGG  R: [Btn]CCCCTTCCTCAACAATTTAA  S: TTTYGTTAGTGGGG | 90 | 19–23 |
| **d**  3 CpGs | NC_000010.11: 128626385–128626583 | A | F: AGTAGGAATGGTAGGAAAATGTATTGT  R: [Btn] CTCTCCTCAACCAAAACTACCC  S: ATTGTTATATGTTAAGTGTAAAAAG | 113 | 01-02 |
| **e**  3 CpGs | NC_000010.11: 129446224–129446519 | A | F: AGGGGGTAGGGATTGGTTT  R: [Btn] AACTTCACTTACCTAAAACTAATAC  S: TTTTAGTTTATA | 120 | 02–03 |
| **f**  63 CpGs | NC_000010.11: 129466880–1129467320 | A | F: GGTTAGGYGTATAGGGTA  R: [Btn] CCRAACCTAAAACAATCTAC  S: GTYGGAGGATTAG | 89 | 11–16 |
| **g**  15 CpGs | NC_000010.11: 129467340–129467540 | A | F/S: GGTGGTAGTTTYGAGTGGTTTTGT  R: [Btn] AAAATCAAAACRACCCCACACCC | 68 | 04–07 |
|  |  | B | F: TTGGGGTTGGTATTTAGAGGTAAG  R: [Btn] CTCCCAAATATTACCCAACCTTTC  S: AGGTAGGGGTTGT | 128 | 12–15+1 |
| **h**  3 CpGs | NC_000010.11: 129604668–129604758 | A | F: GTTGTAGTGGTTTATTGGG  R: [Btn] ATAAAAAACCTTAAAATCACAAC  S: TTTATTTGTATT | 113 | 01–02 |

F: forward primer, R: reverse primer, S: sequencing primer, Y: C/T, R: G/A

**Table S4**. Overview of sample sizes and data availability across all analyses

|  | n | |
| --- | --- | --- |
| Analysis | GB | MBM |
| Methylation | 34 | 20 |
| Methylation/protein | 34 | 20 |
| Methylation/mRNA | 0 | 20 |
| Overall survival | 33 | 17 |

**Suppl Fig 1** Scatter plots illustrating the relationship between CpG methylation levels and MGMT protein expression for all analyzed promoter-, enhancer-, and silencer-associated CpGs in GB and MBM samples.

R-packages:

Wickham et al. (2019). Welcome to the tidyverse. Journal of Open Source Software, 4(43), 1686. <https://doi.org/10.21105/joss.01686>.

Taiyun Wei and Viliam Simko (2021). R package 'corrplot': Visualization of a Correlation Matrix (Version 0.92). <https://CRAN.R-project.org/package=corrplot>

Hadley Wickham (2016). ggplot2: Elegant Graphics for Data Analysis. Springer-Verlag New York. <https://ggplot2.tidyverse.org>

Alboukadel Kassambra (2023). ggpubr: 'ggplot2' Based Publication Ready Plots. R-package version 0.6.0. [https://CRAN.R-project.org/package=ggpubr](https://cran.r-project.org/package=ggpubr)

William Revelle (2024). psych: Procedures for Psychological, Psychometric, and Personality Research. Northwestern University, Evanston, Illinois. R package version 2.4.6. <https://CRAN.R-project.org/package=ggsignif>

Baptiste Auguie. (2017). gridExtra: Miscellaneous functions for “grid” graphics (Version 2.3). <https://CRAN.R-project.org/package=gridExtra>

David Robinson, Alex Hayes and Simon Couch (2022). broom: Convert Statistical Objects into Tidy Tibbles. R package version 1.0.6. <https://CRAN.R-project.org/package=broom>

Winston Chang W (2023). extrafont: Tools for Using Fonts. R package version 0.19. <https://CRAN.R-project.org/package=extrafont>

Hahne F, Durinck S, Ivanek R (2024). Gviz: Plotting data and annotation information along genomic coordinates. R package version 1.54.0. <https://bioconductor.org/packages/Gviz>

Lawrence M, Huber W, Pagès H, Aboyoun P, Carlson M, et al. (2024). GenomicRanges: Representation and manipulation of genomic intervals. R package version 1.62.0. <https://bioconductor.org/packages/GenomicRanges>

Torchiano M (2020). effsize: Efficient Effect Size Computation. R package version 0.8.1. <https://CRAN.R-project.org/package=effsize>
